# Domestic dilemma: when cultivated plants lose their wild side

**DOI:** 10.1093/conphys/coy039

**Published:** 2018-07-09

**Authors:** Alison M Haynes

**Affiliations:** Centre for Sustainable Ecosystem Solutions, School of Biological Sciences, University of Wollongong, NSW, Australia

Can a plant that has been artificially cultivated for centuries survive in the wild? The answer is not a resounding ‘no’, but a new study from China certainly sheds doubt. Yet this question is crucial for conservation efforts worldwide, whether for reintroductions, revegetation, or insurance against future extinction.

The new research investigates the domestication of a plant used in Chinese traditional medicine since the Ming Dynasty (1368–1644). *Glehnia littoralis*, a herb in the carrot family, grows on the east coast of China and is prized for its long taproot that is used to treat lung diseases. The plant is at risk of extinction in the wild because the sandy coastlines it favours are being used for tourism.

Yanxia Pan and colleagues (2018) compared the cultivated plant to its wild relatives to find how it had changed since domestication. They wanted to know how buoyant the seeds were in seawater and how well they germinated after immersion. Combined, these traits are adaptations that are crucial to the plant’s survival in the wild. There, seeds can get swept into the sea and be immersed for some time before being washed up onto a suitable piece of land where, if they are to persist, they must germinate. So, would the cultivated herbs still have those key traits?

While the wild plant *needs* buoyant seeds that will germinate after immersion, these traits are of no advantage to a plant growing far from water. The way evolution operates, individual plants that are well adapted to their environment are more likely to survive and produce seeds that will grow to produce seeds themselves, passing traits from generation to generation as genes.

If wild plants growing by the sea have to cope with recurrent storm surges, they should have genes for buoyancy and immersion. But, if generations of plants grow away from the sea, they may lose these key genes.

**Figure:**
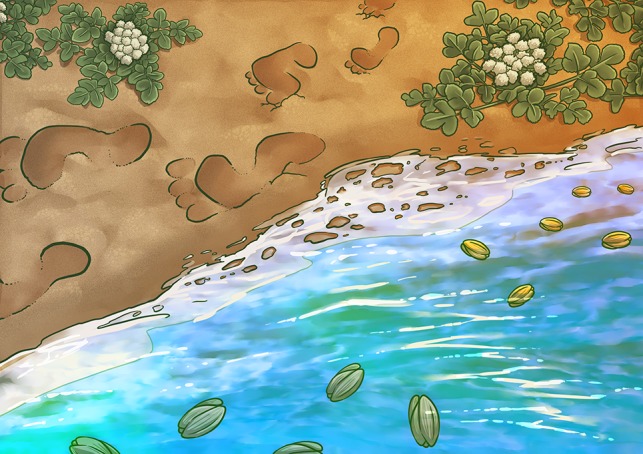


Was this the case with *Glehnia littoralis*? Did inland cultivation cause it to lose its wild traits? The researchers put the plants to the test. The results were clear. Only 30% of seeds from cultivated plants floated compared to almost 100% of their wild cousins'. And, they were dramatically less successful germinating after immersion. That means that if the plants were to disappear from the coast, they could not be replaced with plants from inland populations.

This reminds us that using seeds from cultivated populations could jeopardize conservation efforts. We definitely need to investigate this field further. For example, how quickly could a plant restore key traits if reintroduced? How can we ensure key traits are not lost?

In the meantime, the researchers call for better conservation of the plant’s natural habitat by setting aside reserves. Conserving natural habitat is, by far, the best way to conserve a species. And, the message is clear. Cultivation is not sufficient to protect a species from extinction. We need to make sure we also draw on the strengths of their wild side.

Illustration by Erin Walsh; Email: ewalsh.sci@gmail.com
